# Worrying Presence of Asymptomatic Bacterial Colonisation on Implanted Orthopedic Devices

**DOI:** 10.7759/cureus.68126

**Published:** 2024-08-29

**Authors:** Leonardo Previ, Raffaele Iorio, Mariacarmela Solmone, Daniele Mazza, Fabio Marzilli, Riccardo Di Niccolo, Federico Corsetti, Edoardo Viglietta, Alessandro Carrozzo, Nicola Maffulli

**Affiliations:** 1 Orthopedics and Traumatology, Ospedale Sant'Andrea, Rome, ITA; 2 Microbiology, Ospedale Sant'Andrea, Rome, ITA; 3 Orthopedics and Trauma, Ospedale Santo Spirito, Pescara, ITA; 4 Orthopedics and Traumatology, Ospedale Sant'Andrea, rome, ITA; 5 Trauma and Orthopaedic Surgery, University of Rome, Rome, ITA

**Keywords:** bacterial colonization, sonication, biofilm, orthopedic microbiome, microbiota, microbiome

## Abstract

Background

Bacterial infection after hardware implantation in orthopedic and trauma surgery is devastating, resulting in increased hospital costs and stays, multiple revision surgeries, and prolonged use of antibiotics. The present study aims to determine whether a symbiotic relationship between the human organism and bacteria in hardware implantation may be present, without clinically evident infection.

Materials and methods

We studied explanted devices for microbiological analysis, using the sonication technique, from patients who underwent surgical removal of musculoskeletal hardware for mechanical reasons. None of the patients included in the study had clinical or biochemical signs of infection.

Results

Forty-nine patients were enrolled. Cultures tested positive for bacteria in 42.8% of the 49 patients (21 of 49). In 13 patients, Gram-positive bacteria were isolated, while Gram-negative bacteria were isolated from nine patients. The most frequent bacterial species found was *Pseudomonas aeruginosa*, with six positive cultures (28.5%). Coagulase-negative staphylococci were isolated from ten implants (47%).

Conclusion

A pacific coexistence between humans and bacteria is possible following the implantation of metallic devices for trauma or orthopedic ailments. It is still unclear how strong or unstable this equilibrium is.

## Introduction

Technological developments in medicine have led to implantable devices (pacemakers, catheters, stents, prostheses) that have changed the management of various conditions [1-3]. This is particularly evident in musculoskeletal surgery, where surgical interventions often require implantable devices [4].

Several biomaterials have been developed for orthopedic implants. Among the metals, the alloys most commonly used are titanium, cobalt, and stainless steel, together with polymers [such as polymethyl methacrylate (PMMA) and ultrahigh-molecular weight polyethylene (UHMWPE)] and ceramics (alumina, zirconia, and hydroxyapatite) [5,6].

Implanted devices increase the risk of infection, a most dreaded complication in orthopedic and trauma surgery [7]. Periprosthetic joint infection (PJI) represents one of the most important complications after joint replacement surgery; its incidence varies between 0.5% and 2.2% [8,9]. Fracture-related infection rates after internal fixation vary from 1-2% in closed fractures and rise to 25-30% in the case of open injuries [10,11].

The implant-related infection risk increases because of an immunological deficit at the interface between the implant and the host, which reduces the ability to clear microorganisms from the vicinity of the biomaterial [7]. Once a device has been implanted, host tissue cells compete with bacteria to adhere and colonize the surface of the device [4,7]. Bacterial adhesion represents the first step in implant-related infection, as it can initiate the formation of biofilm, a structured microbial community of bacteria that are attached to a substratum and immersed in a matrix produced by themselves [9]. Biofilms increase antibiotic resistance and offer protection from the host immune system [9,12]. Biofilms modify bacterial phenotypes, especially growth rates and gene expression [12,13].

Detection of microorganisms on biomaterial implants can be difficult because of the presence of biofilm and often requires techniques more advanced than standard cultures. The sonication bath technique allows the identification of bacteria more readily in subclinical infections [12,14-16].

While the presence of bacteria on orthopedic implants in infected patients is well studied [17-20], only two studies investigated microbial colonization of implants in clinically infected patients [21,22].

The present study investigated the presence of bacteria on orthopedic implants in hosts with no suspicion of infection to assess whether and how often non-pathological human-bacteria coexistence occurs. The authors hypothesize the peaceful coexistence between bacteria and implanted hardware, even in patients with no clinical or laboratory signs of infection.

## Materials and methods

Patients who underwent routine removal of non-infected orthopedic devices for trauma and elective surgeries in a university teaching hospital were included in this prospective cohort study from March 2020 to December 2022. All included patients signed the informed consent.

Patients with no clinical signs of infection (swelling, redness, pain, wound dehiscence) or laboratory infection markers, including C-reactive protein (<5 mg/dL), white blood cell count (< 10 × 109/L), procalcitonin (< 0.05 ng/mL), erythrocyte sedimentation rate (< 30 mm/h), and fibrinogen (< 400 mg/dL) at the time of hardware removal, were defined as non-infected and included in the study.

Exclusion criteria were previous surgical site infections, open fractures, pseudarthrosis, and imaging abnormalities which could induce suspicion of implant-associated infections, uncontrolled diabetes, immune depression, systemic use of corticosteroids or immunosuppressive treatment, radiotherapy, and chemotherapy.

A total of 49 patients were enrolled in the study, and 130 devices were sent for sonication, microbiological culture, and, if necessary, antibiogram. The first surgeries (implantation) were performed in different hospitals, but the removal surgery and microbiological investigations were all undertaken in our center.

For each patient, the length of surgery (for both implantation and explantation), complications (for both implantation and explantation), the reason for surgery, the implantation site, the type of implant, the reason for removal, and preoperative antibiotic prophylaxis were analyzed.

At the time of explant surgery, all patients underwent antibiotic prophylaxis with 2 g of cefazolin, according to the hospital guidelines.

Microbiological method

Each implant was removed with separate instruments and placed into a sterile Conformité Européenne (CE) certification-marked leak-proof container. Specimens were transported within 30 minutes from explantation to the microbiological laboratory and immediately processed. More than one implant (two or more devices) was collected for each patient. They were processed separately for the culture analysis of aerobic and anaerobic microorganisms through the addition of brain-heart infusion broth (BHI) and thioglycollate broth (THI) respectively after sonication at 30-40 kHz and 0.22 ± 0.04 W/cm2 for five minutes (BactoSonic; Bandelin Electronic, Berlin, Germany) to favor the detachment of the microbial biofilm; 0.1 mL aliquots of sonicated fluid were plated onto aerobic and anaerobic sheep blood agar plates, which were incubated at 37°C aerobically and anaerobically for two days.

The specimens were incubated in enrichment broth for 14 days and examined visually daily for evidence of growth. Bacterial species were identified with MALDI-TOF MS (Bruker Daltonik, Bremen, Germany), and related antibiograms were performed by standard microbiological methods using an automated system (BD PhoenixTM, BD Diagnostic Systems, Sparks, MD, USA).

Statistical analysis

The results were analyzed using SPSS (IBM Corp. Released 2017. IBM SPSS Statistics for Windows, Version 25.0. Armonk, NY: IBM Corp). A Mann-Whitney U-test for independent samples was performed to compare the paired means of the two measurement groups. P values of < 0.05 were considered significant.

## Results

Forty-nine patients were enrolled in the study. Their mean age was 45 (14-93) years; 26 patients were males (53.06%). The implants were in situ for a median time of 541 (14-4560) days. In 33 of the 49 patients (67.34%), the time in situ was > 1 year. Table 1 reports descriptive statistics of the overall population.

**Table 1:** Descriptive statistics of the overall population WBC: white blood cell; ESR: erythrocyte sedimentation rate; CRP: C-reactive protein; PCT: procalcitonin.

	Min	Max	Mean	Standard dv.
Age (years)	14.0	93.0	45.583	19.9156
Length of first surgery (minutes)	30.0	450.0	97.056	73.4835
Days with implant	14.0	4560.0	542.630	204.5011
WBC count (10 × 10^9^/L)	3.21	15.90	7.0644	2.57770
Neutrophils (%)	7.620	70.600	55.55378	10.463676
Lymphocytes (%)	13.80	47.00	30.7844	7.01822
ESR (mm/h)	1	5	2.8	1.6
CRP (mg/dL)	0	3.2	0.5	1.6
PCT (ng/mL)	0	0.04	0.02	0.02
Serum creatinine (mg/dL)	0.53	1.26	0.94	0.27
Blood urea (mg/dL)	6.00	61.00	25.1591	13.19609

Cultures from implants tested positive for bacteria in 42.8% of the patients analyzed (21 of 49). All positive cultures showed growth within the first 48 hours of incubation. In 13 cases (61% of all positive), gram-positive bacteria were isolated, while gram-negative bacteria were isolated in nine patients (42% of all positive). The bacterium most frequently isolated was Pseudomonas aeruginosa, with six positive cultures (28.5%). In ten patients (47%), coagulase-negative staphylococci were isolated. Table 2 reports the results of the microbiology cultures.

**Table 2:** Bacteria species isolated

Microorganism	N.
Coagulase-negative staphylococci	10
-Staphylococcus epidermidis	4
-Staphylococcus capitis	2
-Staphylococcus warneri	2
-Staphylococcus hominis	1
-Staphylococcus haemolyticus	1
Pseudomonas aeruginosa	6
Stenotrophomonas maltophilia	2
Staphylococcus aureus	1
Ralstonia spp.	1
Micrococcus luteus	1
Bacillus cereus	1

In one patient, the culture was positive for two bacteria: Pseudomonas aeruginosa and Stenotrophomonas maltophilia. The hardware removed were plates, screws, nails, tension bands, Kirschner wires, and devices for graft fixation in anterior cruciate ligament (ACL) reconstruction.

Table 3 shows the comparison analysis between the population with positive and negative culture tests. The only statistically significant difference concerns the percentage of lymphocytes, which was lower, but still within the normal range, in the group of patients with a positive culture.

**Table 3:** Group statistics and analysis WBC: white blood cell; ESR: erythrocyte sedimentation rate; CRP: C-reactive protein; PCT: procalcitonin.

	Positive culture group		Negative culture group		
	Mean	Standard deviation	Mean	Standard deviation	p
Age (years)	46.160	21.2969	46.000	18.5010	0.84
Length of first surgery (minutes)	88.83	41.435	105.28	96.215	1.00
Days with implant	985.583	1099.9507	803.774	922.9734	0.22
WBC count (10 × 10^9^/L)	6.6057	2.37480	7.5441	2.74628	0.16
Neutrophils (%)	56.72696	12.591988	54.32727	7.759675	0.09
Lymphocytes (%)	28.1261	6.33305	33.5636	6.73446	0.01
ESR (mm/h)	2.8	1.6	2.6	1.7	0.67
CRP (mg/dL)	0.4	1.8	0.5	1.1	0.82
PCT (ng/mL)	0.02	0.02	0.02	0.01	1.0
Serum creatinine (mg/dL)	0.8926	0.19184	1.1241	1.32126	0.54
Blood urea (mg/dL)	25.7826	14.54447	24.4762	11.86431	0.96

Table 4 reports the sites of implantation.

**Table 4:** Site of implantation

Site	N. overall	N. positive group
Arm	2	1
Elbow	4	2
Forearm	1	-
Wrist	1	-
Thigh	5	1
Knee	10	4
Ankle	24	12
Foot	2	1

The antibiograms showed one case of methicillin-resistant Staphylococcus aureus, while no other antibiotic resistance was detected among the isolated bacteria.

## Discussion

The main finding of the present study was that bacterial colonies were detected in more than 40% of the patients studied; The presence of bacteria did not produce clinical or laboratory signs of infection in any patient. Implanted material may impact susceptibility to infection, including bacterial adhesion, immune activation, and phagocytosis of bacteria, but its role is still unclear [4].

The reported results show the presence of bacterial colonies on devices implanted in patients who did not present any clinical and laboratory markers of infection; moreover, the patients showed no signs of infection at the last follow-up. The peaceful coexistence of bacteria and humans is well known, the most evident examples being probably the gut [23-25] and the skin microbiomes [26-28]. The literature on the non-pathological coexistence between humans and bacteria in the orthopedic field is, on the other hand, limited.

To our knowledge, only two studies report the coexistence of bacteria and humans on orthopedic implants; Knabl et al. [21] showed a colonization rate of 56% in orthopedic implants in healthy patients, while Fuchs et al. [22] showed a colonization rate of 22%. In the present study, coagulase-negative staphylococci were the most commonly detected microorganisms (47.7%); this finding is in line with Knabl et al.’s and Fuchs et al.’s results; however, the second most represented bacterium was Pseudomonas aeruginosa (28.5%). Fuchs et al. did not detail all the types of bacteria detected. In our cohort, there was only one case of methicillin resistance, while in the rest of the patients, no critical issues related to antibiotic sensitivities were reported; Fuchs et al. and Knabl et al. did not report data about antibiograms.

An analysis of the time between implantation and explantation shows that the hardware remained in place for one patient for only 14 days. This patient (who had his first surgery at another hospital) underwent revision surgery for a secondary fracture-dislocation. The implants were removed, and the osteosynthesis was performed with new devices with no consequences. No risk factors were found in the population examined. The study shows that pacific coexistence between bacteria and humans is possible in an orthopedic context, although it is unclear how stable this equilibrium can be.

There can be multiple critical issues for bacterial contamination and colonization: the local environment (operating room, air, surfaces, surgical equipment, clothing of the medical staff), and bacteria on the patient’s skin and in the patient’s body are significant sources of infectious agents [29].

When comparing the patients with positive vs. negative culture tests, the percentage of lymphocytes was the only statistically significant difference in the haematochemical parameters analyzed. Patients with lower lymphocyte levels (albeit still in the normal range) at the time of explant surgery have a higher probability of bacterial colonization of the implanted devices. The authors are unsure how to interpret this finding; a medium-low lymphocyte level may be a sign of a more permissive immune system that facilitates bacterial coexistence.

In the hypothesis of a symbiotic coexistence, bacterial colonies may modulate the immune response, as happens for intestinal and skin microbiota [30-32]. Unfortunately, we have no data on the lymphocyte count at the time of implantation (first surgery). We are unsure, however, whether this would be of any use, as traumatic events alter lymphocyte count.

Implant removal procedures comprise almost 30% of all planned orthopedic surgeries [33]. Routine implant removal, especially in asymptomatic patients, is highly controversial [34,35]. The results presented raise doubt that these procedures should be performed more frequently; the presence of bacterial colonies, even if well tolerated by the host, does not guarantee that this relationship cannot become pathological and thus produce clinically frank infections.

Such a high percentage of bacterial colonization raises some questions: Should we reconsider antibiotic prophylaxis or at least its length? Which factors regulate the immune response and bacterial activity preventing the development of an infection?

Over the past three years, some authors have introduced the concept of joint microbiota, identifying multiple bacterial colonies within osteoarthritic knees using next-generation sequencing techniques [36,37]. Recent research [38] introduced the concept of knee microbiome profiles: Fernández-Rodríguez et al. demonstrated the presence of bacterial populations with next-generation sequencing tests in both healthy knees and those affected by osteoarthritis (but not infected); different microbiome profiles may play a role in the development of septic arthritis [38].

The presence of bacteria on explanted hardware could be evidence of an implant-related microbiome, although further studies are needed to further investigate this issue.

Considering the present results, it may be possible that more implants should be removed at the end of the healing process. In clinical practice, however, it should be considered that routine removal of asymptomatic hardware is technically more demanding than its implantation, and carries more risk of complications, including post-operative infections.

Also, two-stage surgery for those patients who need hardware to be removed to undergo prosthetic procedures should be considered.

This study has several limitations. For example, we are aware of the small sample size of the present study, arising from the strict inclusion criteria. Next-generation sequencing analysis or electron microscopy scanning could have been performed, but these technologies were not available in our setting. Also, implant locations differed, and no conclusions about the site-related risk could be drawn. Follow-up is relatively short, but none of the patients returned to our attention complaining of problems to date. Also, preoperative antibiotic prophylaxis was executed prior to hardware removal. However, the evidence in the literature regarding the effect of preoperative antibiotic prophylaxis on culture examination is quite controversial [39-44] and the current study was designed as observational and therefore did not change our surgical practice.

## Conclusions

Even asymptomatic implants can show evidence of bacterial colonization. In the absence of signs and symptoms of infection, it is unclear how these findings impact clinical practice. The presence of bacteria on explanted hardware could be evidence of an implant-related microbiome, although further studies are needed to further investigate this issue. Until recently, the presence of bacteria in the orthopedic setting was associated almost exclusively with a pathological condition; the authors believe it is important to focus attention on the mechanisms and equilibrium of a symbiotic relationship between bacteria and humans.
